# An Easy and Fast Method for Production of Chinese Hamster Ovary Cell Line Expressing and Secreting Human Recombinant Activin A

**DOI:** 10.22074/cellj.2020.6580

**Published:** 2019-10-14

**Authors:** Hassan Rassouli, Ali Sayadmanesh, Siamak Rezaeiani, Zahra Ghezelayagh, Mohammad Reza Gharaati, Tahamtani Yaser

**Affiliations:** 1. Department of Molecular Systems Biology, Cell Science Research Center, Royan Institute for Stem Cell Biology and Technology, ACECR, Tehran, Iran; 2. Department of Medical Laser, Medical Laser Research Center, Yara Institute, ACECR, Tehran, Iran; 3. Department of Stem Cells and Developmental Biology, Cell Science Research Center, Royan Institute for Stem Cell Biology and Technology, ACECR, Tehran, Ira; 4. Department of Developmental Biology, University of Science and Culture, Tehran, Iran; 5. Interdisciplinary Department for Diabetes, Obesity and Metabolism, Cell Science Research Center, Royan Institute for Stem Cell Biology and Technology, ACECR, Tehran, Iran

**Keywords:** Activin A, Cell Proliferation, CHO Cells, Embryonic Stem Cells, Recombinant Protein

## Abstract

**Objective:**

Growth factors are key elements of embryonic stem cell (ESC) research. Cell line development in eukaryotes is a time-consuming procedure which usually takes 12-18 months. Here, we report an easy and fast method with which production of Chinese hamster ovary (CHO) cells that express and secrete recombinant Activin A, as a major growth factor in endo/mesoderm differentiation of embryonic stem cells is achieved within 3-4 weeks.

**Materials and Methods:**

In this experimental study, we cloned human Activin A into the pDONR/Zeo gateway entry vector using the BP reaction. Activin A was subcloned next into the pLIX_403 and pLenti6.3/TO/V5-DEST destination vectors by the LR reaction. The result was the production of constructs with which 293T cells were finally transfected for virus production. CHO cells were transduced using viral particles to produce a cell line that secretes the His6- Activin A fusion protein.

**Results:**

We developed a quick protocol which saves up to 3-4 weeks of time for producing recombinant proteins in CHO cells. The recombinant cell line produced 90 mg/L of functional Activin A measured in human ESC line Royan H5 (RH5), during *in vitro* differentiation into meso-endoderm and definitive endoderm.

**Conclusion:**

Our results showed no significant differences in functionality between commercial Activin A and the one produced using our novel protocol. This approach can be easily used for producing recombinant proteins in CHO.

## Introduction

Growth factors though play important roles in stem cell research, are regarded as one of the most expensive components of culture media. Activin A has a wide range of biological activities in hematopoietic mesoderm induction, reproductive physiology, bone remodeling, and most importantly, in neural cell differentiation (1-4). Activin A plays a critical role in the initial step of stem cells differentiation towards endoderm precursors such as lung cells, hepatocytes and pancreatic progenitors (5-7). Huge progress was made worldwide concerning the differentiation of stem cells towards insulinsecreting beta-like cells. A highly efficient method of endoderm cell production is, therefore, necessary to gain high numbers of pancreatic progenitors and endocrine cells for further clinical applications (8, 9). Activin A, as a growth factor is a homo-dimeric polypeptide (27 kDa molecular weight) which is a member of the transforming growth factor (TGF)-â superfamily )10(. The precursor protein has 426 amino acids but after completion of the maturation process, only 116 amino acids Gly311-Ser426 remain. amino acid Cys390 from the two chains attach each other by a disulfide bond to make a homo-dimeric Activin A (11).

The emergence of induced pluripotent stem cells (iPSCs) and trans-differentiation have increased the hope for using recombinant transcription factors associated with cell-penetrating peptides to facilitate the conversion of different cell types toward specific cell types. In this regard, the following differentiation approaches has previously been reported: conversion of human embryonic stem cell (hESC) into cardiomyocyte using ISL1 protein (12), hESC into dopaminergic neurons using recombinant LMX1A factor (13), human fibroblasts into dopaminergic neural progenitor-like cells using recombinant Yamanaka factors (14) and human fibroblasts toward cardiomyocytelike cells via recombinant Yamanaka factors (15); nevertheless, the growth factors are still key elements in the production of different cell types.

Recombinant growth factors are being commercially produced in both prokaryotes and eukaryotes. The most popular protein expression systems are bacteria (*E. coli*), yeast (*S. cerevisiae*), insect or mammalian (HEK293 and CHO cells) systems. Factors like: time, amount of needed protein, ease of handling, disulfide bonds formation and type of post-translational modifications (PTM) determine the type of expression system and host used to produce recombinant proteins. Technically, production of recombinant proteins in *E. coli* is simpler and could be done in a significantly shorter period of time (16, 17). Expression of some proteins still needs to be done in eukaryotes because some expressed proteins in *E. coli* are not properly folded and they may require PTM such as glycosylation, lipidation, methylation and acetylation (18), or eukaryotic cells chaperons for correct folding (19) or tertiary/ quaternary structure formation despite its higher costs and longer time period requirement. Also, for protein-protein interaction (PPI) studies, recombinant proteins must be expressed in their original cell so the researchers will have a better understanding of proteins network (20).

CHO cells were derived from a CHO about 61 years ago in Theodore Puck’s lab (21) and became the first choice for therapeutic and non-therapeutic recombinant proteins production in eukaryotic cells (22, 23). Nowadays, globally, hundreds of billions of Dollars are annually spent on the production of recombinant proteins in CHO cells (24). This further highlights the importance of producing recombinant proteins in CHO cells. One of the major steps in producing recombinant proteins in eukaryotic cells is the development of stable cell lines which produce sufficient amount of proteins. Typically, this step may take up to 6-12 months (25, 26).

Here, we report the development of a quick protocol which takes 3-4 weeks to develop CHO cell line with acceptable yield. In addition, expression of functional human Activin A was measured by sodium dodecyl sulfate polyacrylamide gel electrophoresis (SDS-PAGE), western blotting, and MTS assay; and hESC differentiation into definitive endoderm was also investigated.

## Materials and Methods

### Isolation of Activin A cDNA

In this experimental study, according to previously published data (27), 20 day old embryoid bodies (EB) derived from human ESCs express Activin A mRNA. EBs total RNA was isolated using TRIzol (Sigma- Aldrich, USA) according to the manufacturer’s protocol. The first strand of cDNA was synthesized using SuperScript III reverse transcriptase (Invitrogen, USA), an oligo dT primer, and 2 ìg of purified total RNA. For Activin A amplification, primers were designed to amplify nucleotides 931-1281 (Accession # NM_002192.2) corresponding to Gly311- Ser426 amino acids (Accession # P08476). Generated cDNA was amplified using below-mentioned primers:

AttB1-Ig κ1: 5´-GGG GAC AAG TTT GTA CAA AAA AGC AGG CTG CCG CCA CCA TGG AGA CAG ACA CAC TCC TGC TAT GGG TAC TGC TGC TCT GGG TTC CAG GTT CCA CTG GTG- 3'

Ig κ1-His: 5'- GTT CCA GGT TCC ACT GGT GAC CAT CAC CAC CAC CAT CAT-3'

His-Activin: 5´-CAT CAC CAC CAC CAT CAT GGC TTG GAG TGT GAT GGC-3´

AttB2-activin: 5´-GGG GAC CAC TTT GTA CAA GAA AGC TGG GTC TAT GAG CAC CCA CAC TC-3´

Primers contained Igκ1 signal peptide, 6 His tag, and gateway attachment site B1 (AttB1) and AttB2 sequences used for protein secretion, purification, and quick cloning, respectively. Also, a stop codon was included in the sequence to terminate the translation reaction. For fragment amplification, pfx DNA polymerase (Invitrogen, Carlsbad, CA, USA) and Mastercycler® Gradient PCR (Eppendorf Netheler-Hinz GmbH, Germany) were used. Amplification was done using 3 tandem PCR reactions as follows: The first polymerase chain reaction (PCR) included pre-incubation at 95˚C for 4 minutes; 10 cycles at 95˚C for 30 seconds, 60˚C for 30 seconds, and 68˚C for 40 seconds with His-Activin and AttB2-activin primers; The second PCR was comprised of 10 cycles at 95˚C for 30 seconds, 60˚C for 30 seconds, and 68˚C for 40 seconds with Ig κ1-His and AttB2-activin primers; and the third PCR included 30 cycles at 95˚C for 30 seconds, 60˚C for 30 seconds, and 68˚C for 40 seconds, followed by incubation with AttB1-Ig κ1 and AttB2-activin primers at 68˚C for 8 minutes. PCR products were analyzed by electrophoresis on a 1% agarose gel, stained with ethidium bromide and examined under ultraviolet (UV) light.

### Construction of the pENTER/Activin A entry clone

The resultant PCR product was cloned into the pDONR/ Zeo gateway entry vector using the BP clonase according to the supplier�fs directions (Invitrogen, USA). The recombinant pENTER/Activin A entry clone was transferred into Library Efficiency® DH5α™ Competent Cells (Invitrogen, USA) by the heat shock method as described by the manufacturer. Clones were cultured in Luria-Bertani (LB) broth overnight and plasmid extraction was performed using the AccuPrepR Plasmid Mini Extraction Kit (Bioneer, Korea). Recombinant vectors were examined by PCR using the M13-F and Activin -R primers which generated an amplicon of about 650 bp. DNA sequencing of the inserted segment was done using M13-F: 5'-GTA AAA CGA CGG CCA GT-3' and R: 5'-AGC GGA TAA CAA TTT CAC ACA GGA-3' primers.

### Construction of the pLIX_403/Activin A and pLenti6.3/ TO/V5-DEST/Activin A expression vectors

A pENTER /Activin A entry clone construct with correct direction and sequence was chosen for the LR reaction in which, Activin A was transferred from the entry clone into the pLIX_403 and pLenti6.3/TO/V5-DEST destination vectors according to the manufacturer’s instructions (Gateway® Technology, Invitrogen, Carlsbad, CA, USA). Products of LR reaction were transferred into Library Efficiency® DH5α™ Competent Cells (Invitrogen, Carlsbad, CA, USA) by the heat shock method as described by the manufacturer and recombinant expression vectors were confirmed by PCR. Also, we cloned the GFP and RFP markers in pLenti6.3/TO/V5-DEST and pLIX_403, respectively to test the transduceability of CHO cells as well as vectors’ elements proper function.

### Viral particle preparation

Viral particle preparation was performed as described previously (28). The 293T cells were seeded in 10-cm cell culture dishes. Once cells reached 70% confluency, they were transfected with Lipofectamine 3000 according to the supplier¡¯s manual. Recombinant lentiviral particles were harvested every 24 hours for 2 days, filtered, aliquoted and kept at -80˚C for future uses.

### Activin A-secreting cell line establishment

The CHO- DG44 cells were grown in Dulbecco’s modified Eagle’s medium/F12 (DMEM-F12) medium (Gibco, USA) with 1% fetal bovine serum (FBS, Gibco, USA). Cells were seeded in T25 culture dishes and the frozen viruses were added to culture medium. Addition of viral particles was repeated 24 hours later while exchanging the medium. Cells were kept for another 24 hours and then, replated at a ratio of 3:1 in new T25 dishes for antibiotic selection and stable cell line development. Antibiotics, blasticidin, and puromycin were used for pLenti6.3 TO V5-DEST and pLIX_403, respectively, for 10 days.

### Recombinant Activin A expression and secretion

As both pLenti6.3 TO V5-DEST and pLIX_403 are Tet-on vectors, 5-10 μg/ml doxycycline was applied to the culture medium for inducing Activin A expression in generated cell lines. To increase the yield, the temperature was set at 32˚C. Culture medium was refreshed every day and finally collected and stored at -80˚C for protein purification.

### Recombinant fusion protein purification

The cell debris was precipitated by centrifugation at 14,000 g for 5 minutes, and the supernatant was used for purification. Recombinant His6-Activin A was purified by the Ni-NTA Fast Start Kit (Qiagen, USA). The column was washed with 10 ml of washing buffer [20 mM Tris- HCl (pH=8.0), 150 mM NaCl and 25 mM imidazole] to remove non-specifically bound proteins. His6-Activin A that remained on the column was eluted using 1 ml elution buffer which contained 250 mM imidazole in 3 separate fractions. In each step, 20 ìl sample was preserved for further analysis by SDS-PAGE.

The concentration of the purified protein was determined by the Bradford method. Recombinant Activin A was dissolved in a proper storage buffer, filter-sterilized (0.2 μm), distributed into vials (10 μg per vial), lyophilized, and stored at -80˚C for future functional bioassays. The recombinant Activin A was named "homemade Activin A".

### SDS-PAGE and mass spectrometry analysis

Identical volumes of different elution fractions were mixed with 5:1 volume of 5X loading buffer [1 M Tris- HCl (pH=6.8), 10% w/v SDS, 0.05% w/v bromophenol blue, 50% glycerol, and 200 mM β-mercaptoethanol] and heated at 95˚C for 5 minutes before analysis by SDS-PAGE using a 12% (w/v) separating gel followed by staining with 0.1% Coomassie brilliant blue R-250. Bands of interest were excised from the SDS-PAGE gel and samples were analyzed by liquid chromatography coupled with tandem mass spectrometry (LC-MS/MS) at Sydney University.

### Western blotting

Western blot analysis was performed as described previously (29). Briefly, proteins were separated by 12% SDS-PAGE electrophoresis at 100 V for 2 hours using a Mini-PROTEAN 3 electrophoresis cell (Bio-Rad, Hercules, CA, USA) then transferred to a polyvinylidene difluoride (PVDF) membrane by wet blotting (Bio-Rad, Hercules, CA, USA). Membranes were blocked for 1 hour using 5% bovine serum albumin (BSA, Sigma- Aldrich, USA), and incubated for 1.5 hours at room temperature (RT) (30) with the following primary antibodies [anti- His6 (provided with Ni-NTA Fast Start Kit (Qiagen, USA) 1:5000]. Membranes were rinsed 3 times (15 minutes each) with Phosphate-buffered saline Tween-20 (PBST, 0.05%) and incubated with the peroxidase-conjugated secondary antibody [anti-mouse (Millipore, 1:6000)], for 1 hour at RT. The blots were visualized using Sigma detection reagents (Sigma- Aldrich, USA) and films were scanned by a densitometer (GS-800, Bio-Rad, USA).

### Biological analysis of homemade Activin A by MTS assay

Biological analysis was performed using the method described by Phillips and colleagues (31). For Activin A, examination of dose-dependent inhibition of the proliferation of mouse plasmacytoma cell line (MPC-11) which is routinely employed by companies like Sigma and thermo fisher for testing recombinant Activins, was done. In this assay, rates of inhibition of cell proliferation were assessed using the Cell Titer 96 Non-Radioactive Cell Proliferation MTS Assay Kit (Promega, UK) according to manufacturer¡¯s manual. Briefly, after testing the viability of the cell lines, cells were plated in 96- well, flat-bottom plates and allowed to attach for a few hours. Serial dilutions of recombinant homemade Activin A and commercial Activin A from Sigma (0-10 ng/ml) were prepared in 96-well flat-bottom plate in SFM. For the control group, the cells were cultured in the absence of Activin A. Subsequently, the cells were added to the wells of a 96-well plate and incubated for 3 days at 37˚C in a humidified, 5% CO_2_ atmosphere. After this period, cells viability was assessed by 3-(4,5-dimethylthiazol- 2-yl)-5-(3-carboxymethoxyphenyl)-2-(4-sulfophenyl)- 2H-tetrazolium (MTS). In this assay, 20 μl of the MTS reagent was added into each well and cells were incubated at 37˚C for 3 hours. The absorbance was detected at 490 nm by a microplate reader. All the experiments were repeated three times.

### Human embryonic stem cell culture

hESC line RH5 (passage 36) was obtained from the Royan Stem Cell Bank (Royan Institute, Iran) and cultured in ES cell maintenance medium, on Matrigel (Sigma- Aldrich, USA)-coated plates as previously reported (32, 33). ES cell maintenance medium contained DMEM-F12 plus GlutaMAX (Gibco, USA) supplemented with 20% knockout serum replacement (KoSR, Invitrogen, USA), 1% insulin-transferrin-selenium (ITS, Invitrogen, USA), 0.1 mM non-essential amino acids (NEAAs, Invitrogen, USA), 1% penicillin/streptomycin (Invitrogen, USA), 0.1 mM â-mercaptoethanol (Sigma-Aldrich, USA), and 100 ng/ml basic fibroblast growth factor (bFGF, Royan Biotech, Iran). Human ES cells were grown in 5% CO_2_ atmosphere with 95% humidity. The medium was changed every other day. For maintenance of the cells, they were passaged every 7 days at a 1:4-1:6 split ratio using collagenase IV (0.5 mg/ml, Invitrogen, USA): dispase (1 mg/ml, Invitrogen, USA) at a ratio of 1:1

### Generation of human embryonic stem cell-derived endoderm

Human ESC-derived definitive endoderm differentiation of stem cell colonies began on day 4 of stem cell culture. In the first step, to achieve the meso-endoderm, the hES medium was changed to RPMI-1640 plus GlutaMAX (Invitrogen, USA) supplemented with 1% penicillin/streptomycin, 0.1 mM NEAAs, 0.5% BSA, 2 ìM Chir99021 (Stemgent, USA) and 100 ng/ml Activin A (Sigma-Aldrich, USA) for the control group, or 25, 50,100 and 200 ng/ml homemade Activin A for the 4 experimental groups. After 24 hours, in order to reach the definitive endoderm, Chir99021 was removed and the cells were treated for 48 hours with 50 mM ascorbic acid, 5 ng/ml bFGF and 100 ng/ml Activin A for the control group, or 25, 50,100 and 200 ng/ml homemade Activin A for the experimental groups. Before each differentiation step, cultured cells were washed in Dulbecco’s phosphate-buffered saline with calcium and magnesium (DPBS, Gibco, USA). It must be mentioned that all the experimental groups were treated with homemade Activin A lot 111 and 112 batches.

### Immunocytostaining analysis

Immunocytofluorescence staining was performed using a previously described method (34). Briefly, cells were fixed with 4% paraformaldehyde (PFA, Sigma-Aldrich, USA) for 20 minutes, permeabilized using 0.1% Triton X-100 for 10 minutes, blocked with 10% secondary antibody host serum in 0.5% BSA for 1 hour at 37˚C, and finally incubated with goat anti-human SOX17 antibody (R&D Systems, USA) diluted 1:200 in 0.5% BSA, at 4˚C overnight. For negative controls, primary antibodies were omitted and a similar staining procedure was followed. Cells were subsequently washed with PBST and incubated with diluted (1:700) donkey antigoat IgG-Alexa Fluor® 546 antibody (Invitrogen, USA) for 1 hour. Cell nuclei were stained with 4', 6-diamidino- 2-phenylindole (DAPI, Sigma-Aldrich, USA) for 1 minute and afterward observed under a fluorescence microscope (BX51, Olympus, Japan) equipped with Olympus DP72 digital camera for imaging. For each group, six 40X frames were captured and the percentage of positive cells observed in these frames was calculated by ImageJ. The percentage was expressed as mean ± standard deviation (SD).

## Results

### Cloning of Activin A cDNA and construction of the entry clones and the expression vectors

**Fig 1:**
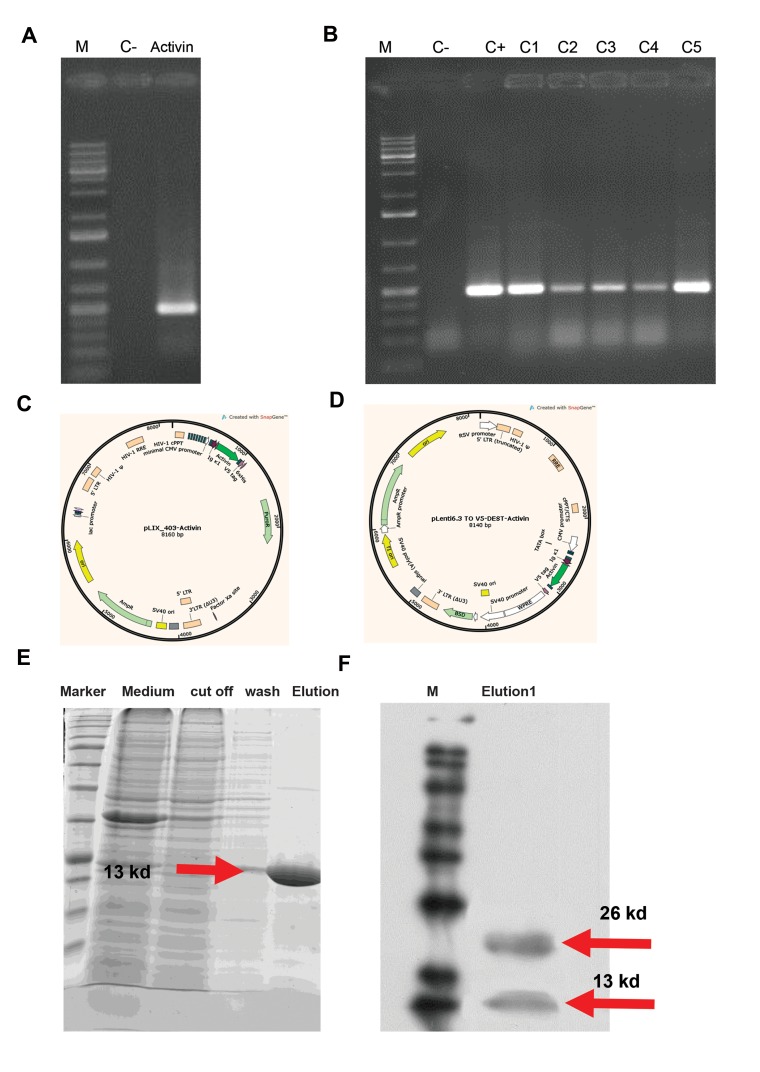
Activin A cloning and expression confirmation. PCR analysis of amplified Activin A, SDS-PAGE analysis and western blotting of produced Activin A. **A.** The expected 499-bp product of Activin A was amplified by PCR using primers that added Ig κ1/His tag/AttB1 to 5'end and AttB2 to 3' end, **B.** Colony PCR products of Activin A for five clones (C1-C5), **C, D.** pLIX_403/Activin & pLenti6.3/TO/V5-DEST/Activin constructs map, **E.** SDS-PAGE analysis of produced Activin A. Recombinant his-tag-Activin A was successfully expressed, secreted and purified. The purified proteins showed the expected size band (13 kD), and **F.** Western blotting of Activin under non-reduced condition. Here, 13 and 26 kD proteins show monomer and dimer forms of Activin, respectively. These results confirm that at least 50% of secreted Activin is in dimer form and possibly folded correctly. M; Size marker, C-; Negative control, C+; Positive control, PCR; Polymerase chain reaction, and SDS-PAGE; Sodium dodecyl sulfate polyacrylamide gel electrophoresis.

The 499-bp Activin A /Ig κ1/His tag/AttB1&2 gene was amplified (Fig .1A) from hESC cDNA and subsequently cloned in a pDONR/Zeo gateway entry vector using the BP reaction to produce a pENTER/Activin A entry clone. The recombinant entry clone was transferred into Library Efficiency® DH5á™ Competent Cells and as a result, tens of clones appeared on the next day. Since the gateway cloning method has low false-positive results, all clones were confirmed by PCR analysis and five clones were randomly selected for further analysis (Fig .1B). DNA sequencing results showed that four out of five clones had no mutation; a clone with no mutation was used for further LR reactions with pLIX_403 (Fig .1C) and pLenti6.3 TO V5- DEST (Fig .1D). The pENTER/Activin A entry clone 1 and pLenti6.3/TO/V5-DEST and pLIX_403 destination vectors were separately used for constructing the expression clone using the LR reactions and were transferred into Library Efficiency® DH5α™ Competent Cells. Five clones out of tens of clones were randomly tested by colony PCR for both destination vectors. We observed that all clones were positive for Activin A insertion, indicating that the LR reactions were 100% efficient. For both expression vectors, a similar clone was selected for virus production and cell line establishment.

### More than 50% of purified homemade Activin A showed dimer form

The CHO pLIX_403/Activin A (Fig .1C) or pLenti6.3/ TO/V5-DEST/Activin A (Fig .1D) stable cell lines were grown in DMEM-F12 medium and induced by addition of 5-10 ìg/ml doxycycline. Media were collected every day and expressed fusion proteins were purified by immobilized metal affinity chromatography (IMAC) on a nickel 2+ column using 25 mM imidazole, which eliminated the majority of contaminating proteins in the flow and through the washing steps. The fusion protein was obtained in the 250 mM imidazole fractions (Fig .1E). The identities of the purified fusion proteins were confirmed by trypsin digest and LC/MS/MS. The MS results indicated that our fusion proteins matched the Activin A protein (Accession No. NP_999193.1; data not shown). Western blotting under non-reduced conditions showed 13 and 26 kD proteins indicating monomer and dimer homemade Activin A, respectively (Fig .1F). This result confirms that at least 50% of the secreted Activin A is in dimer form and are most likely folded correctly.

### pLenti6.3/TO/V5-DEST/GFP and pLIX_403/RFP vectors could express high levels of recombinant protein

**Fig 2:**
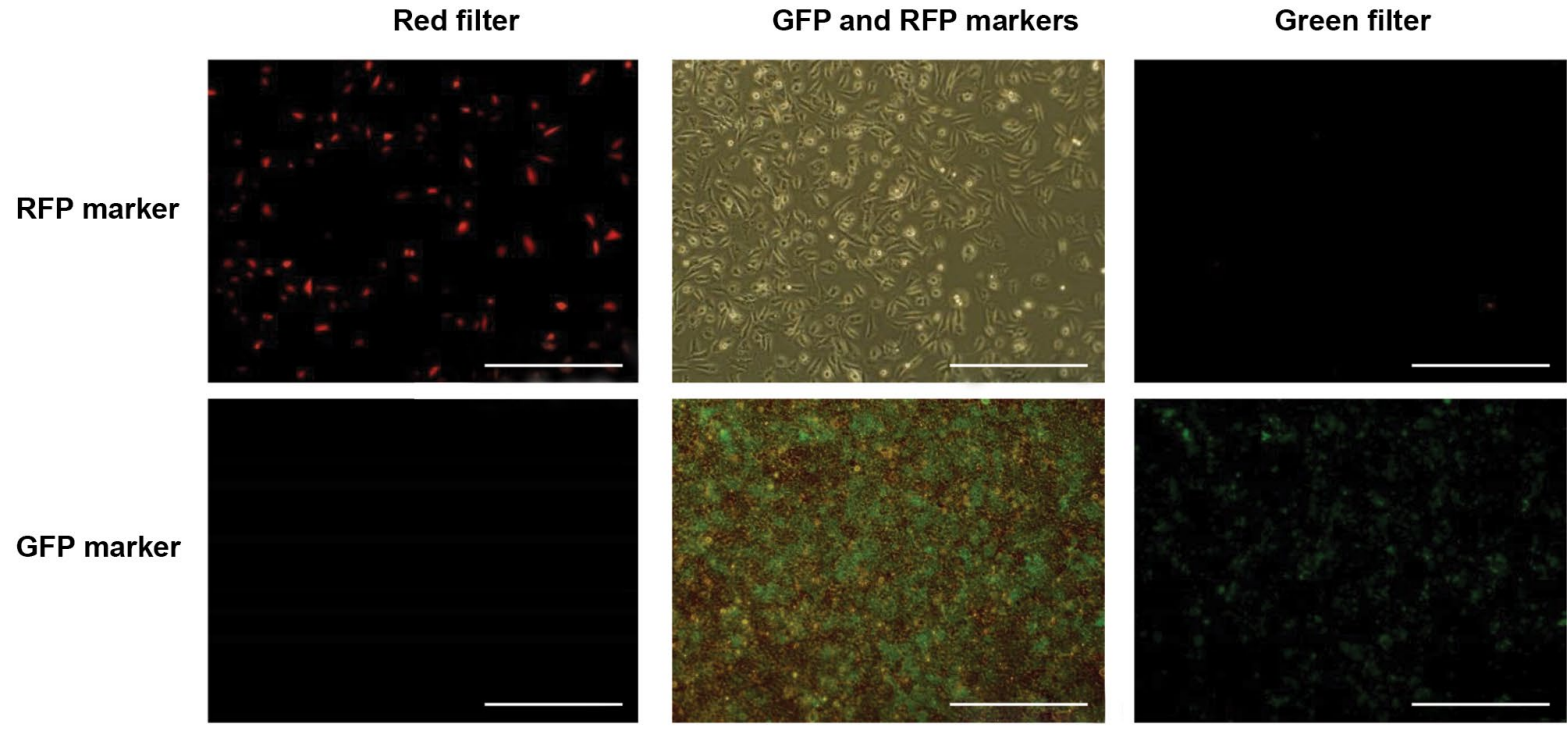
Fluorescent microscopy imaging results confirm that viral particles produced by pLenti6.3/TO/V5-DEST/GFP and pLIX_403/RFP expression vectors, can efficiently transduce CHO cells and express high levels of inserted genes (scale bar: 200 μm).

As mentioned earlier, pLenti6.3/TO/V5-DEST/GFP and pLIX_403/RFP were used to test the proper function of the vector, viral particle preparation protocol and also, CHO cells transfect ability. Fluorescent microscopy imaging results (Fig .2) showed that both pLenti6.3/TO/V5-DEST/ GFP and pLIX_403/RFP expression vectors could express high levels of inserted genes. In addition, both vectors were functional in CHO cells and viral particles produced in 293T cells could transduce CHO cells very efficiently.

### Homemade Activin A could inhibit the proliferation of MPC-11 cells

**Fig 3:**
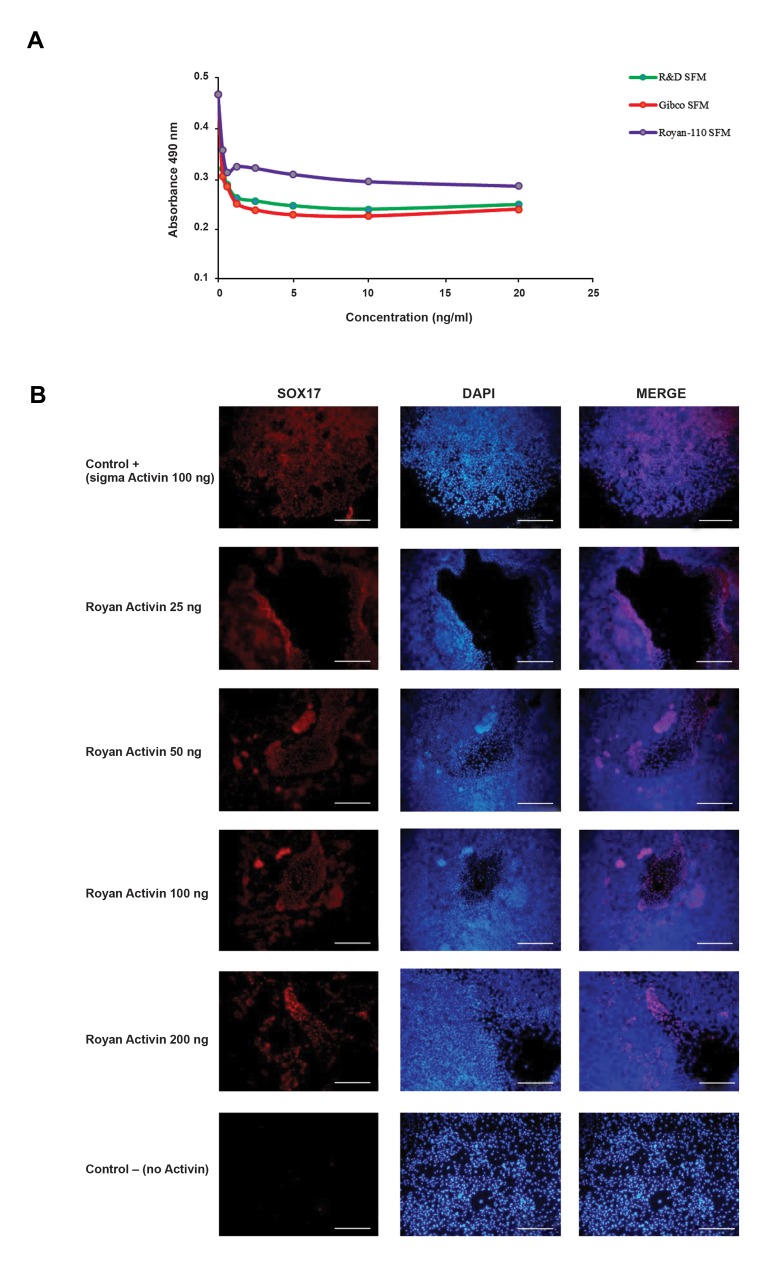
The biological activity of the homemade Activin confirmed it is comparable with commercial Activin’s. **A.** Dose-dependent inhibition of MPC-11 proliferation assessed by MTS. The results indicated that Activin A at concentrations up to 20 ng/ml, can inhibit the proliferation of MPC-11 cells in a dosedependent manner and its activity is about 70% of that of the commercial Activin’s (Gibco and R&D) and **B.** Differentiation of human ES cell into definitive endoderm cells. The cells treated with 25, 50, 100 and 200 ng/ml homemade Activin lot 111 showed 13, 23, 35 and 43% SOX17 expression, respectively while cells treated with Activin A from Sigma, expressed 76% SOX17 marker (scale bar: 200 μm).

The biological activity of the recombinant Activin A with respect to its ability to dose-dependently inhibit the proliferation of MPC-11 was assessed by MTS assay. The results shown in Figure 3A indicated that Activin A at concentrations up to 20 ng/ml, can inhibit the proliferation of MPC-11 cells in a dose-dependent manner. The activity of homemade Activin A is about 70% of that of the commercial Activin’s (Gibco and R&D).

### Homemade Activin A-treated human embryonic stem cells expressed high levels of SOX17

To evaluate the efficiency of endoderm induction by homemade Activin A, the expression of definitive endoderm marker, SOX17, was analyzed on differentiation day 4 in human ESCs (RH5 cell line). Immunofluorescent staining showed the expression of SOX17 in both control and treated groups (Fig .3B). The control group treated with commercial Activin A, markedly expressed SOX17 marker (76.3%), while the groups treated with homemade Activin A expressed lower percentages of the endodermal marker. The cells treated with homemade Activin A lot 111 at concentrations of 25, 50, 100 and 200 ng/ml showed 13, 23, 35 and 43% of SOX17 expression, respectively. Cells treated with 25, 50, 100 and 200 ng/ml homemade Activin A lot 112 revealed 30, 37, 20 and 33% of SOX17 expression, respectively.

## Discussion

 In the present study, we cloned a cDNA encoding human Activin A into the pDNOR/Zeo gateway entry vector using the BP reaction, then, into pLIX_403 and pLenti6.3/TO/V5-DEST destination vectors by using the LR reaction. We used Gateway Technology as it is a rapid, highly-efficient technique and suitable for cloning and sub-cloning of several target genes simultaneously. This technology provides a wide range of destination vectors for different applications. Also any vector could be converted into gateway compatible destination vectors with single step ligation reaction. The pLIX_403 and pLenti6.3/TO/ V5-DEST vectors have a strong promoter which allows production of high levels of recombinant proteins under the control of doxycycline and has tight control over the expression induction under desired conditions. We used CHO cells which are the prominent eukaryotic cells used for protein expression. CHO cells glycosylation pattern is highly similar to that observed in humans.

As previously shown by several studies (35), routine and standard approaches take about 12-18 months for cell line development, while our experiment was completed within 3-4 weeks. This allows researchers to test tens of variables to get the optimum conditions and elements needed for best protein expression and possibly industrial applications. The produced recombinant Activin A had correct folding with no inclusion body and its production was markedly cost-effective. We assessed the functionality of homemade Activin A during hES cell line RH5 differentiation into meso-endoderm and definitive endoderm. We also demonstrated that homemade Activin A that was used in this study is of high quality compared with commercial Activin A. This paves the way for costeffective commercial production of homemade Activin A and substantial reduction in experimental costs especially in the fields of stem cell research and cell therapy.

## Conclusion

 Our results indicated a little difference in functionality between in-house generated and commercialized Activin A where, this shortcoming could be addressed in future. The availability of large quantities of recombinant Activin A would greatly facilitate mouse and human pluripotent stem cell differentiation cultures.

## References

[1] Ying SY, Zhang Z, Furst B, Batres Y, Huang G, Li G (1997). Activins and activin receptors in cell growth. Proc Soc Exp Biol Med.

[2] Smith JC, Price BM, Green JB, Weigel D, Herrmann BG (1991). Expression of a Xenopus homolog of Brachyury (T) is an immediate-early response to mesoderm induction. Cell.

[3] Fathi A, Mirzaei M, Dolatyar B, Sharifitabar M, Bayat M, Shahbazi E (2018). Discovery of novel cell surface markers for purification of embryonic dopamine progenitors for transplantation in parkinson’s disease animal models. Mol Cell Proteomics.

[4] Fathi A, Hatami M, Vakilian H, Han CL, Chen YJ, Baharvand H (2018). Quantitative proteomics analysis highlights the role of redox hemostasis and energy metabolism in human embryonic stem cell differentiation to neural cells. Mol Cell Proteomics.

[5] Farzaneh Z, Najarasl M, Abbasalizadeh S, Vosough M, Baharvand H (2018). Developing a cost-effective and scalable production of human hepatic competent endoderm from size-controlled pluripotent stem cell aggregates. Stem Cells Dev.

[6] Firth AL, Dargitz CT, Qualls SJ, Menon T, Wright R, Singer O (2014). Generation of multiciliated cells in functional airway epithelia from human induced pluripotent stem cells. Proc Natl Acad Sci USA.

[7] Rezania A, Bruin JE, Arora P, Rubin A, Batushansky I, Asadi A (2014). Reversal of diabetes with insulin-producing cells derived in vitro from human pluripotent stem cells. Nat Biotechnol.

[8] Pagliuca FW, Millman JR, Gürtler M, Segel M, Van Dervort A, Ryu JH (2014). Generation of functional human pancreatic β cells in vitro. Cell.

[9] Schulz TC, Young HY, Agulnick AD, Babin MJ, Baetge EE, Bang AG (2012). A scalable system for production of functional pancreatic progenitors from human embryonic stem cells. PLoS One.

[10] Xia Y, Schneyer AL (2009). The biology of activin: recent advances in structure, regulation and function. J Endocrinol.

[11] Stamler R, Keutmann HT, Sidis Y, Kattamuri C, Schneyer A, Thompson TB (2008). The structure of FSTL3.activin A complex.Differential binding of N-terminal domainsinfluences follistatin-type antagonist specificity. J Biol Chem.

[12] Fonoudi H, Yeganeh M, Fattahi F, Ghazizadeh Z, Rassouli H, Alikhani M (2013). ISL1 protein transduction promotes cardiomyocyte differentiation from human embryonic stem cells. PLoS One.

[13] Fathi A, Rasouli H, Yeganeh M, Salekdeh GH, Baharvand HJMb (2015). Efficient differentiation of human embryonic stem cells toward dopaminergic neurons using recombinant LMX1A factor. Mol Biotechnol.

[14] Mirakhori F, Zeynali B, Rassouli H, Salekdeh GH, Baharvand H (2015). Direct conversion of human fibroblasts into dopaminergic neural progenitor-like cells using TAT-mediated protein transduction of recombinant factors. Biochem Biophys Res Commun.

[15] Ghazizadeh Z, Rassouli H, Fonoudi H, Alikhani M, Talkhabi M, Darbandi-Azar A (2017). Transient activation of reprogramming transcription factors using protein transduction facilitates conversion of human fibroblasts toward cardiomyocyte-like cells. Mol Biotechnol.

[16] Rassouli H, Tabe Bordbar MS, Rezaei Larijani M, Pakzad M, Baharvand H, Salekdeh GH (2013). Cloning, expression and functional characterization of in-house prepared human basic fibroblast growth factor. Cell J.

[17] Rassouli H, Nemati S, Rezaeiani S, Sayadmanesh A, Gharaati MR, Salekdeh GH (2013). Cloning, expression, and functional characterization of in-house prepared human leukemia inhibitory factor. Cell J.

[18] Walsh G, Jefferis R (2006). Post-translational modifications in the context of therapeutic proteins. Nat Biotechnol.

[19] Hartl FU (1996). Molecular chaperones in cellular protein folding. Nature.

[20] Yousefi M, Hajihoseini V, Jung W, Hosseinpour B, Rassouli H, Lee B (2012). Embryonic stem cell interactomics: the beginning of a long road to biological function. Stem Cell Rev.

[21] Tjio JH, Puck TT (1958). Genetics of somatic mammalian cells.II.Chromosomal constitution of cells in tissue culture. J Exp Med.

[22] Wurm FM (2004). Production of recombinant protein therapeutics in cultivated mammalian cells. Nat Biotechnol.

[23] Ashall F, Sullivan N, Puck TT (1988). Specificity of the cAMP-induced gene exposure reaction in CHO cells. Proc Natl Acad Sci USA.

[24] Aggarwal RS (2014). What’s fueling the biotech engine-2012 to 2013. Nat Biotechnol.

[25] Sleiman RJ, Gray PP, McCall MN, Codamo J, Sunstrom NA (2008). Accelerated cell line development using two‐color fluorescence activated cell sorting to select highly expressing antibody‐producing clones. Biotechnol Bioeng.

[26] Wurm FM (2004). Production of recombinant protein therapeutics in cultivated mammalian cells. Nat Biotechnol.

[27] Fathi A, Hatami M, Hajihosseini V, Fattahi F, Kiani S, Baharvand H (2011). Comprehensive gene expression analysis of human embryonic stem cells during differentiation into neural cells. PLoS One.

[28] Ghasemi-Kasman M, Hajikaram M, Baharvand H, Javan M (2015). Micro- RNA-mediated in vitro and in vivo direct conversion of astrocytes to neuroblasts. PLoS One.

[29] Fathi A, Pakzad M, Taei A, Brink TC, Pirhaji L, Ruiz G (2009). Comparative proteome and transcriptome analyses of embryonic stem cells during embryoid body‐based differentiation. Proteomics.

[30] Hotchko M, Robert P (2018). Recent market status and trends of fractionated plasma products. Ann Blood.

[31] Phillips DJ, Brauman JN, Mason AJ, de Kretser DM, Hedger MP (1999). A sensitive and specific in vitro bioassay for activin using a mouse plasmacytoma cell line, MPC-11. J Endocrinol.

[32] Fathi A, Rasouli H, Yeganeh M, Salekdeh GH, Baharvand H (2015). Efficient differentiation of human embryonic stem cells toward dopaminergic neurons using recombinant LMX1A factor. Mol Biotechnol.

[33] Faradonbeh MZ, Gharechahi J, Mollamohammadi S, Pakzad M, Taei A, Rassouli H (2012). An orthogonal comparison of the proteome of human embryonic stem cells with that of human induced pluripotent stem cells of different genetic background. Mol Biosyst.

[34] Tahamtani Y, Azarnia M, Farrokhi A, Sharifi-Zarchi A, Aghdami N, Baharvand H (2013). Treatment of human embryonic stem cells with different combinations of priming and inducing factors toward definitive endoderm. Stem Cells Dev.

[35] Lai T, Yang Y, Ng SK (2013). Advances in mammalian cell line development technologies for recombinant protein production. Pharmaceuticals (Basel).

